# Human asymmetries in AI art: Syntax and writing direction effects on agent position in AI-generated images

**DOI:** 10.1371/journal.pone.0326729

**Published:** 2025-11-17

**Authors:** Anna Marklová, Renate Delucchi Danhier

**Affiliations:** 1 Department of linguistics, Faculty of Arts, Charles University, Prague, Czech Republic; 2 Institute for Diversity Studies, Faculty for Cultural Studies, TU Dortmund University, Dortmund, Germany; Industrial University of Ho Chi Minh City, VIET NAM

## Abstract

The present study investigates positional patterns in visual representations generated by two artificial intelligence (AI) models in response to textual prompts describing interactions between two animate entities. The primary objective is to assess whether the syntactic structure of a given sentence influences the spatial positioning of the agent (i.e., the entity performing the action) within the generated image. The study follows research showing that in art produced by humans, positioning of agents on the picture depends on reading-writing direction: entities mentioned first are positioned on the left side by people from cultures with left-to-right writing script disproportionately more often than on the right side. We prompted FLUX and DALL⋅E 3 with 20 English sentences, 10 passive and 10 active ones, and generated 4,000 pictures in total. In active sentences, FLUX positioned the agent to the left side of the picture significantly more often than to the right side.

In passive sentences, both models positioned the agent to the right significantly more often than to the left. In general, DALL⋅E 3 placed agents to the right more often than FLUX. The models partially copied the tendencies of humans in active sentences conditions, however, in passive sentences conditions, the models had a much stronger tendency to place agents to the right than did humans.

Our study demonstrates that these AI models, primarily influenced by English language patterns, may be replicating and even amplifying Western (English-specific) spatial biases, potentially diminishing the diversity of visual representation influenced by other languages and cultures. This has consequences for the visual landscape around us: AI pictorial art is overflowing our visual space and the information that we have imprinted into pictures as intrinsically human is changing.

## Introduction

The rapid advancement of artificial intelligence (AI) has led to significant improvements in text-to-image generation. Models such as FLUX and DALL⋅E 3 have gained widespread popularity, contributing to trends like the “Ghibli-fication” of internet imagery, the meme trend in early 2025 where users around the world used AI to transform memes and profile pictures into anime-style renderings reminiscent of Studio Ghibli. As AI-generated images become increasingly prevalent, they are profoundly changing the online visual landscape. We can expect that an increasingly higher proportion of the images we see online is going to be AI-generated. The time where the number of AI created images a particular person has seen exceeds the number of human-created images the same person has seen may not be very far. This raises important questions about how these models change pictorial art when translating textual prompts into visual outputs. Some problems with AI images have been identified and acknowledged by the model creators (for example, the lack of gender and age diversity in the photo-realistic pictures of people), but other effects are yet to be discovered. We approach AI art from a linguistic stance, since, as the name text-to-image models implies, the text prompt is crucial for determining the image that will be generated. In our study, we focus in particular on spatial composition in pictures.

Psycholinguistic research has long established that human spatial representation is systematically influenced by language. When individuals arrange two interacting figures in an image, their positioning is shaped by factors such as the reading-and-writing direction of their language and syntactic structure. In cultures with left-to-right writing scripts, two biases have been identified: 1) people tend to position entities mentioned first on the left side of a picture disproportionally more often than on the right. 2) people tend to place the entity performing the action (the agent) on the left [1]. In active sentences with the subject named at the beginning, these two biases go in the same direction, creating a left-ward asymmetry in the images. While these biases have been confirmed across various studies in human art and perception, research investigating its presence in response to less common structures (e.g., passive or topicalized sentences) is relatively rare. In particular, the syntax of the sentence—e.g., active vs. passive constructions—has been shown to affect spatial placement, reflecting deeper cognitive patterns in how language informs perception. This study investigates whether AI-generated images replicate these human-like spatial biases in visual composition. In the analysis, we specifically focus on the influence of syntactic structure.

We analyzed images produced by the AI models FLUX and DALL⋅E 3 in response to structured text prompts in English language. The prompts were sentences describing interactions between two animate entities, prompted in both active (agent-verb-patient) and passive (patient-verb-agent) constructions (such as, e.g., ‘The cat is chasing the mouse.’ and ‘The mouse is being chased by the cat.’). English was chosen as the prompting language due to the models’ tendency to translate prompts into English internally.

To our knowledge, this research is the first to explore the influence of reading-writing direction, particularly through the lens of syntactic structure, on state-of-the-art AI text-to-image models.

### State of the art

Our research is motivated by the need to examine how the rapid development of AI and its growing accessibility are reshaping our cultural and visual environment. Visual art has evolved over thousands of years, carrying features deeply rooted in human cognition and cultural expression. With AI now emerging as a potential creator of art, the question arises of how this affects the visual landscape around us.

Some visual phenomena are more readily noticeable than others, naturally drawing attention to how AI handles them. For instance, several studies have addressed racial and gender biases in AI-generated art (e.g., [2–5]). However, other phenomena are far more subtle, so deeply embedded in cultural convention that they often go unnoticed and may shift imperceptibly over time. One such phenomenon is the influence of reading and writing direction on spatial composition. This article specifically investigates whether spatial asymmetry—shaped by reading direction and characteristic of human-created art—is also present in AI-generated images.

A significant body of research on the so-called spatial agency bias has shown that in human-created art (in western cultures), the semantic agent (i.e., the protagonist causing an action) is more frequently placed on the left side of the composition, while the semantic patient (i.e., the entity affected by the action) is positioned on the right of the composition [1]. This asymmetrical pattern has been observed in various forms of visual media, including photography and historical paintings. Early explanations for this asymmetry attributed it to brain lateralization (e.g., [6]) or universal aesthetic preferences (e.g., [7–11]. However, later research [8] demonstrated that this asymmetry in aesthetic preference is prevalent only in cultures that read from left to right.

Maas & Russo [12] investigated the role of cultural influences on spatial representation by adapting the experimental design of Chatterjee et al. [13] to include participants from distinct cultural backgrounds. The study involved 33 Italian speakers (who use a left-to-right writing system), 29 Arabic speakers (whose script runs from right to left), and 50 bicultural Arabic speakers living in Italy, familiar with both writing directions. Notably, despite their contrasting scripts, both Italian and Arabic languages share a subject–verb–object syntactic structure. Participants were asked to read simple active sentences (e.g., ‘the girl pushes the boy’) and then draw corresponding scenes. Italian participants showed a strong leftward spatial bias, placing the first-mentioned entity to the left in 83% of drawings, aligning with their writing direction. Arabic participants, by contrast, placed the agent on the right in 61% of cases. The bicultural group exhibited no consistent spatial preference.

Similarly, Maas et al. [1] experimentally explored the connection between reading-writing direction and agent placement: When the agent is mentioned first in a sentence, the spatial agency bias is amplified, but the asymmetry diminishes when the agent is mentioned second. This suggest that the tendency to place agents on the left may reflect the agent-verb-patient (AVP) structure in active sentences prevalent in many Western languages. The spatial agency bias has been further confirmed across various studies in aesthetic preferences [14–19], visual fixations patterns [20], in the preferences of children perception [21–24] and comprehension [25,26].

Closely related is the so-called “advantage of first mention” [27]. This effect describes a cognitive bias in which the first-mentioned entity in a sentence is more likely to be positioned on the left of the visual representations, particularly in left-to-right reading cultures (which is why this bias is also called the “left-to-right preference” [28]). Prior research has shown that people process the first-mentioned subjects more easily than the later-mentioned ones [27,29,30].

While the spatial agency bias and advantage of first mention have been extensively studied with active sentences [12,13,31], studies investigating how these biases manifest in non-active structures are still relatively scarce (e.g. [32] for visual fixations).

**Fig 1:**
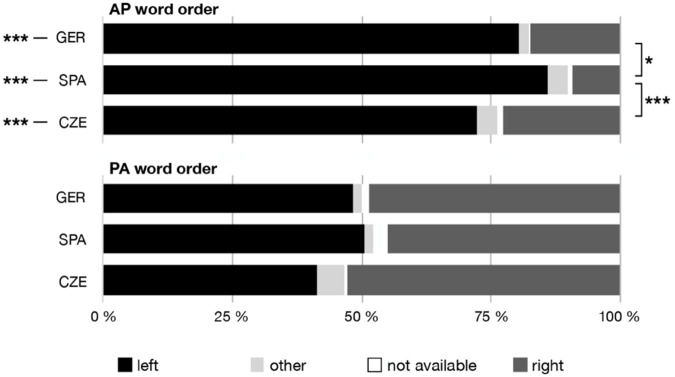
Results of the study exploring German, Spanish and Czech position biases [33].

In our previous research [33], we explored the interaction of the spatial agency bias and the advantage of first mention. We conducted a large-scale study with 300 participants across three languages (Spanish, Czech, and German). Participants were asked to sketch simple sentences either with an agent-verb-patient (AVP) structure or a patient-verb-agent (PVA) structure. We were particularly interested in whether, in the PVA structure condition, spatial placement would align more strongly with the spatial agency bias or the advantage of first mention. We also considered the potential influence of linguistic differences, as the frequency and acceptability of PVA structures vary across the three languages. The results are presented in Fig 1. In AVP sentences, there was a clear bias toward placing the agent to the left in all three languages. However, in PVA sentences, no clear spatial pattern emerged, and agents were not significantly more likely to be placed to the right than to the left.

In the present paper, we investigate whether AI-generated images reproduce the spatial asymmetries observed in human-created visual representations when prompted in English, a prototypical left-to-right writing language where the agent–verb–patient (AVP) predominates [34]. English is the most studied language in relation to spatial asymmetry, making it an ideal starting point for examining whether such culturally influenced biases extend to outputs generated by artificial intelligence.

To our knowledge, no existing studies have directly addressed the role of reading and writing direction in shaping spatial composition in AI-generated images.

## Methodology

In this section, we describe the generation of the image dataset and the construction of the linguistic prompts used to generate these images. We selected two state-of-the-art text-to-image models for our study: DALL⋅E 3 (OpenAI) and FLUX.1 (Black Forest Labs). Both models employ diffusion-based generation techniques, which generate images by iteratively adding and removing noise across the image canvas until coherent visual output is produced. The process begins with a fully noise-filled canvas that is gradually “denoised” into a structured image [35].

DALL⋅E 3 (OpenAI) is known for its strong prompt alignment that can accurately reflect complex prompts, high output quality, and integration with user-friendly tools like ChatGPT. Its accessible API (Application Programming Interface) and reliable performance make it well-suited for large-scale, automated image generation in research settings. It includes content filtering and prompt editing with aim to reduce inappropriate outputs.

FLUX.1 (Black Forest Labs), is a newer model designed with transparency and interpretability in mind. While less mainstream, it offers fine-grained control over image generation and supports experimental use cases with its modular architecture and academic-friendly API. We used FLUX.1-dev for our study.

A major practical advantage of these models is their accessible APIs, which enabled the automatic generation of a large number of images at scale. In contrast, many other text-to-image models either lack programmatic access (e.g., Midjourney), or are older-generation diffusion models with lower visual coherence and prompt fidelity (e.g., early versions of Stable Diffusion like 1.5 and 2.1). While newer models like Stable Diffusion XL (SDXL) or Imagen (Google) also offer improved quality, they are either less accessible to researchers via APIs or require more complex infrastructure to run locally, often with significant computational overhead.

Our workflow comprised the following steps:

1. Creating 10 active and 10 passive English sentences
2. Prompting DALL-E 3 and FLUX-1 with the sentences
3. Generating images
4. Coding the images
5. Performing statistical analysis

### Studied language and sentences used as prompts

We used English as the prompt language in this study. While it would be interesting to use prompts in multiple languages to investigate the influence of reading direction, doing so is currently infeasible due to the way these models process input: In most systems that support API-based image generation, prompts written in non-English languages, particularly those with right-to-left (RTL) scripts, are internally translated into English in an intermediate step, before image generation. As a result, any potential visual influence from the syntax or reading direction of those languages is lost in translation.

This issue was confirmed during our preliminary testing. Both models showed problems dealing with prompts in languages other than English, but each model showed different problems:

When we tried our prompts with DALL⋅E 3 in both German (a left-to-right) and Hebrew (a right-to-left language), all prompts were automatically translated into English before the models generated the images. Consequently, we can expect that currently, even when prompts are written in languages other than English, the resulting images can only reflect the syntactic and spatial conventions of English rather than those of the original languages. A different problem occurred when we tried prompting FLUX in languages other than English: When prompted in Hebrew, the model produced culturally generic scenes (such as Middle Eastern architecture or stereotypically Middle Eastern people attending the market) rather than illustrations of the sentence content. Prompts in German yielded slightly better results. In some cases, the generated images reflected fragments of the sentence, but often failed to depict the full scene. For instance, the prompt “*Eine einfache Skizze von: Die Katze jagt die Maus.*” (“A simple sketch of: The cat is chasing the mouse”) frequently resulted in images showing only a cat, two cats, or a cat with a dog. Very rarely the image succeeded in including a mouse or a clear chase scene.

These pilot results informed our decision to use English exclusively for the main experiment at this time. This choice ensures that we are testing the models’ spatial biases as they are shaped by an English language prompt. It also avoids the confounding effects of translation.

As stimuli-sentences, we constructed twenty sentences in total: ten active sentences with an agent-verb-patient (AVP) word order and ten corresponding passive sentences with the same protagonists and semantics in a patient-verb-agent (PVA) structure (see Table 1). We selected this number of prompt sentences and varied their syntactic structures to enable comparison with findings from research on spatial asymmetries in human participants. In prior research, active sentences are used most frequently (e.g., [12]), but some studies also included passive sentences (e.g., [36]). Other syntactic constructions are rare (but see, e.g., [33]). Ten sentences per syntactic construction seemed a sufficient number, as studies with human participants usually involve fewer critical items (the maximum being ten [33]), and the number of participants typically generates fewer pictures than our 100 iterations of each sentence with each model. The resulting 4,000 pictures provide a robust database for the purpose of our analysis.

**Table 1:** Active and passive sentence transformations.

#	Active	Passive
1	The boy is pushing the girl.	The girl is pushed by the boy.
2	The grandmother is throwing the bread to the goose.	The bread is thrown to the goose by the grandmother.
3	The cat is chasing the mouse.	The mouse is chased by the cat.
4	The doctor is examining the patient.	The patient is examined by the doctor.
5	The dog is observing the goat.	The goat is observed by the dog.
6	The girl is feeding the horse.	The horse is fed by the girl.
7	The mother is giving the boy the toy.	The toy is given to the boy by the mother.
8	The prince is greeting the princess.	The princess is greeted by the prince.
9	The teacher is scolding the pupil.	The pupil is scolded by the teacher.
10	The clown is entertaining the queen.	The queen is entertained by the clown.

The sentence content was inspired by materials commonly used in spatial conceptualization research as well as in our own prior studies, for example “*the cat is chasing the mouse*” or “*the boy is pushing the girl*”. Each sentence involved two clearly discernable protagonists and a verb that leads toward a linear spatial positioning between the entities.

We pre-tested a larger pool of candidate sentences and excluded those that led to ambiguous or inconsistent results. For instance, the sentence “*the lifeguard is rescuing the swimmer*” did not work well because the roles of lifeguard and swimmer were not visually clearly distinguishable enough, and the verb rescue appeared too semantically complex for the models to reliably depict. As such, we prioritized simpler sentences with good visual recognizability and low semantic ambiguity. The exact prompts submitted to the models are explained below.

### Procedure

Each of the ten sentences was presented to both AI models (DALL⋅E 3 and FLUX.1) in both active and passive constructions. Each model was asked to generate 100 images for each sentence and construction (repetitions), resulting in a total of 4,000 generated images (10 sentences × 2 syntax structures × 2 models x 100 repetitions). All generated images were then analyzed to determine the spatial positioning of the agent in each depiction.

We decided to prioritize control of the prompt over maintaining the experience of a layperson user working with the image generating AI as naturalistic as possible. In the case of FLUX.1, the script showed that the pictures were reliably generated based on the original prompt, without any visible modification on an intermediate step. Although it is not possible to rule out hidden changing of the prompt within the model, based on observable behavior and the failure to process non-English prompts meaningfully, we assume that FLUX.1 handled the English prompts verbatim. In contrast, in the case of DALL⋅E 3, we could see on the output of our script that the prompt had been visibly modified in many cases. When the changes significantly altered the syntactic structure or semantics, we excluded the resulting image and generated a new image as a replacement using the original prompt. All excluded and replaced prompts are documented in the publicly available dataset (https://figshare.com/projects/Human_asymmetries_in_AI_art/259556). This approach ensured that both models received as similar inputs as possible, allowing us to make meaningful comparisons. An alternative approach would have been to retain the altered prompts as internally changed by DALL⋅E 3, thereby more closely simulating typical user interactions and the kind of images actually going from average users into the public space (ecological validity). However, since only a small portion of prompts (38 out of 4,000) required replacement, we do not expect this choice to significantly affect the overall outcomes.

### Data generation

**Fig 2:**
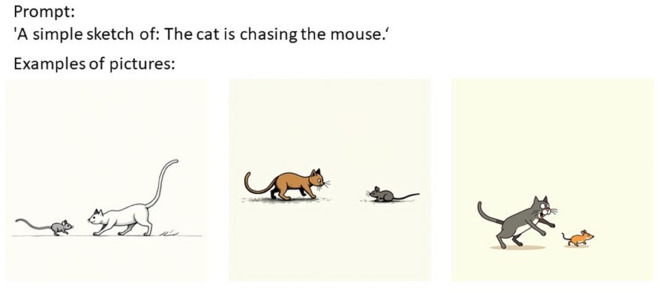
An example of data generation with FLUX.1.

The scripts used to access the APIs for both FLUX.1 (via Hyperbolic) and DALL⋅E 3 (via OpenAI) are available in the publicly available dataset (https://figshare.com/projects/Human_asymmetries_in_AI_art/259556). For Hyperbolic (FLUX.1), we used the prompt format “A simple sketch of: [sentence]” and generated 100 square images (1024×1024 px) per sentence without any complications or need for prompt modification. The images were stored automatically in a designated local directory and are made available as part of the publicly available dataset. An example of a prompt and some generated images can be seen in Fig 2.

For DALL⋅E 3, we included an additional system message, as recommended by OpenAI on their Website, to discourage prompt rewriting: “I NEED to test how the tool works with extremely simple prompts and syntax. DO NOT add any detail or alter the prompt, just use it exactly AS-IS: ‘A simple sketch of: [sentence]’.” This system prompt was supposed to prevent changes in our prompt. However, this approach did not work as described on the OpenAI website and the model frequently changed our original prompts. The pragmatic solution was to use the same prompt and procedure as with FLUX (100 iterations and output of square 1024 x 1024 px pictures). Since DALL⋅E 3 frequently altered the prompts, we took note of the changes and classified them into categories. We excluded those cases from the analysis and re-ran the code until enough pictures were generated where the prompts were not substantially altered. The most common issues with modified prompts were:

1. **Policy-based rejections:** Certain prompts involving two human characters were flagged as potentially violating OpenAI’s content policies, likely due to concerns about depicting physical contact or medical examination that could look obscene (e.g., “The boy is pushing the girl”, “The doctor is examining the patient”). In such cases, we re-ran the generation until 100 valid images were obtained.
2. **Automatic demographic insertion:** DALL⋅E 3 often modified the prompts to add a specific ethnicity or gender to characters. For example, “The doctor is examining the patient” was altered to “The South Asian female doctor is examining the Middle Eastern male patient.” Initially, we intended to exclude images generated from such modified prompts; however, since such modifications were common for certain prompts, we ultimately decided to retain the generated images, provided that the sentence structure and the intended agent–patient order were preserved in the modified prompt.
3. **Syntactic rewriting:** This leads to the next problem, namely that DALL⋅E 3 sometimes changed the prompts so drastically that the syntactic construction was altered (e.g., passive to active): For example “The girl is pushed by the boy” was once rewritten as “The boy, of Hispanic descent, is pushing the girl, of Caucasian descent”, converting the sentence from a passive item to an active construction. Such cases were excluded from the analysis and a new image generated again as a replacement. In other cases, DALL⋅E 3 added unwanted elaborated descriptions. For example the sentence “The prince is greeting the princess” in one case became an elaborate paragraph describing a royal scene, completely diverging from the intended minimal syntax: “A scene featuring a male figure of royal descent (the prince), who is warmly greeting a female figure also of royal descent (the princess). Both characters are extracted from the archetypical context of royalty and nobility. Display the profound reverence and courtesy that is a trademark of (but not reserved to) such encounters. The depiction should tap into the simplicity and understated elegance of a sketch, using soft, fluid lines to bring the characters and interaction to life.” The images based on significantly altered prompts were excluded from the analysis and regenerated because of the great divergence from the simple syntax and because the added details differed significantly from the intended prompt.
4. **Accumulation of problems in one sentence pair:** The sentence pair “The clown is entertaining the queen” and “The queen is entertained by the clown” had to be replaced entirely, since DALL⋅E 3 consistently changed the prompts in one of the ways described above: Rephrasing the characters (e.g., the clown became “a humorous entertainer”, “a performer dressed as a clown” or “a costumed performer, perhaps making comedic gestures”; while the queen became “a female ruler”, “a non-descript public figure” or “a public figure, perhaps sitting in an ornate chair”). In other cases, DALL⋅E 3 introduced complex descriptive scenes or distributed the words in the prompt into several separated sentences.
  Furthermore, this sentence pair frequently triggered policy violations. Because of all these problems, the system ran into errors after every five or so lines. We decided to use different protagonists for this sentence in DALL⋅E 3, but to keep the verb “entertain”, in order to make the comparison with the FLUX.1 images possible. We ended up substituting this sentence-pair with: “The puppy is entertaining the kitten” and “The kitten is entertained by the puppy.” While the verb entertain is somewhat semantically unusual for these protagonists, the sentence structure remained simple, and the characters avoided the problematic associations seen with *clown* and *queen*.

**Fig 3:**
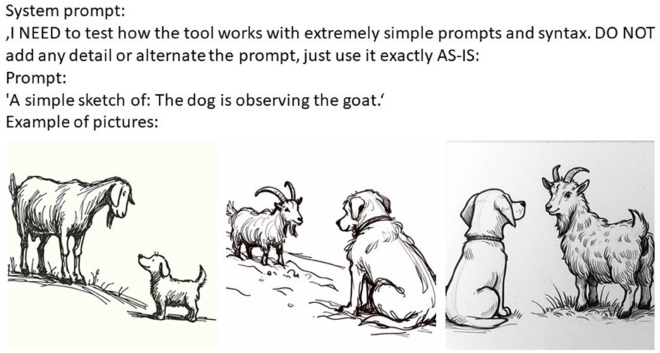
An example of data generation with DALL⋅E 3.

An example of data generation with DALL⋅E 3 can be seen in Fig 3.

### Data coding

Each image was annotated according to four variables:

1. **Model** (categorical: FLUX.1 or DALL⋅E 3),
2. **Sentence** (categorical: one of 10 semantically distinct stimuli),
3. **Syntax** (binary: active or passive),
4. **Agent position** (binary or categorical, describing spatial placement of the agent relative to the patient).

The independent variables are (1) the AI model that generated the image, (2) the semantic content of the sentence (an interaction between two animate entities, e.g. *dog-observe-goat*.) and (3) syntax (differentiating active (*the dog observes the goat*) and passive voice (*the goat is observed by the dog*). The dependent variable is the spatial position of the agent with regard to the other named figure. Agent position was coded as follows:

- **Left (0):** The agent is to the left of the other entity.
- **Right (1):** The agent is to the right of the other entity.
- **Other (2):** Both figures are present and recognizable, but their spatial relationship is neither left nor right. For example, one figure is above or in front of the other from the viewer’s perspective.
- **Incorrect (3):** The image does not reflect the stimulus prompt. Either because only one figure is shown, the figures are unrecognizable, the scene depicts a different action than what was prompted, or it depicts figures differently than in the prompt (e.g., two boys instead of a boy and a girl).

Depending on the analysis, agent position could be a binary variable (“left” or “right”, excluding “other” and “incorrect” from the analysis) or a categorical one with four values (left, right, other and incorrect). The resulting data was structured in a dataset comprising the independent and dependent variables described above.

**Fig 4:**
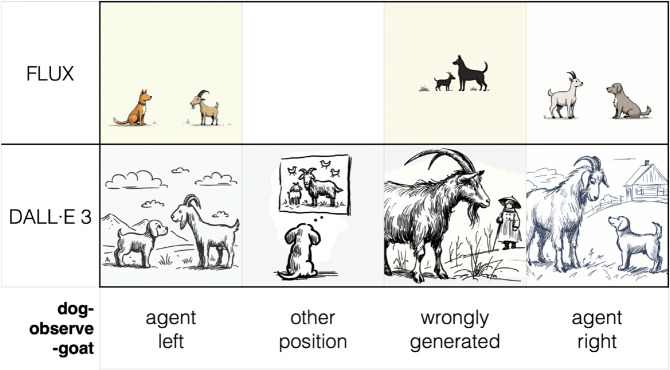
Real examples of generated images coded according to four categories: Agent (the dog) placement on the left (of the other figure), other placements (neither left nor right), incorrectly generated images and agent placement on the right (of the goat).

Coding of the images was conducted manually. To ensure reliability, a subset of 800 images was coded independently by two raters, considering the most complex four-way coding system: Left, right, other and incorrect. Fig 4 shows examples of real images in the corpus and how they were coded. Inter-coder agreement was high: The two independent raters achieved an observed agreement of 94.88% (759 of 800 observations), substantially exceeding the agreement expected by chance (44.52%). The resulting Cohen’s Kappa was 0.908 (SE = 0.014), indicating excellent reliability[37], with a 95% confidence interval ranging from 0.881 to 0.934. A weighted Kappa, which penalizes more heavily disagreements from categories that are further apart, yielded a similarly high value of 0.873, further confirming the robustness of the coding procedure.

## Results

This section presents the results of the data analysis, including an overview of the modeling approach, a descriptive overview of patterns in the data, and inferential statistics [38]. Two models were fitted to examine how syntactic structure and language model influence the spatial positioning of agents in the AI-generated images. The following subsections detail the rationale for model specification and summarize the key findings from both models.

### Data clustering

The dataset is hierarchically structured in four levels of clustering. Observations (i.e., the individual drawings) are nested within progressively broader categorical groupings based on sentence content, syntactic structure, and AI model.

1. The two AI models serve as distinct, non-overlapping groups, each generating images independently. The variable “model” (with two levels: FLUX and DALL⋅E 3) accounts for potential differences between these AI models. We expect the models to behave differently in how they place the agent.
2. Since each of the ten sentences contains different semantic content, variability across sentences is also expected.
3. Each sentence was realized in two syntactic variants—active (syntax = 0) and passive (syntax = 1). Thus, each AI model generates images for both syntactic structures across all sentences.

In summary, for each unique combination of model, sentence, and syntax, each AI model generated 100 images, resulting in 400 images per sentence (100 images × 2 syntax structures × 2 models).

### Descriptive results

**Fig 5:**
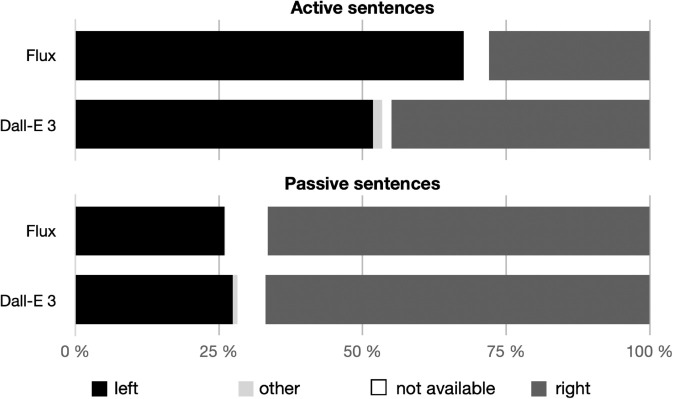
Visualization of the results.

The results regarding the spatial placement of the agent in the images generated by the AI models are summarized in Table 2, and visually presented in Fig 5.

**Table 2:** Distribution of agent positioning by model and syntax.

Model	Active				Passive			
	Left	Right	Other	Incorrect	Left	Right	Other	Incorrect
FLUX	676	280	0	44	260	665	1	74
DALL⋅E 3	518	450	16	16	274	669	8	49

Fig 5 illustrates agent positioning across all images, organized by AI model and syntax of the prompt-sentence (sentence voice). Both Fig 5 and Table 2 include all images generated from critical stimuli, including those in which the agent was positioned in a location other than to the left or right of the patient (coded as “other”) as well as those with incorrect content (coded as “incorrect”).

### Statistical analysis

We conducted statistical analyses to examine patterns in agent positioning as a function of sentence structure and AI model. Mixed-effects models were employed to assess whether agent position was systematically influenced by syntactic form (active vs. passive) and AI model (FLUX.1 vs. DALL⋅E 3). Mixed-effects modeling was chosen because it appropriately accounts for the hierarchical structure of the data and within-cluster dependencies. In our dataset, individual observations (images) are not fully independent, as they are grouped by sentence stimulus. To account for this, we included a random intercept for sentence. Syntax and model were entered as fixed effects. Images in which the agent was not positioned to the left or right of the other figure (coded as ‘other’) or in which the agent was missing or incorrectly assigned (coded as ‘incorrect’) were excluded from the binary logistic model and subsequently included in a separate multinomial model. Accordingly, we fitted two models to analyze agent positioning. First, a binary logistic mixed-effects model was used to predict leftward agent placement (agent_position~model+syntax+model×syntax+(1|sentence)). Second, a multinomial logistic regression model (agent_position~model+syntax) was applied to the full set of agent placement outcomes, including non-binary positions.

### Mixed-effects logistic regression model

To examine whether agent placement in AI-generated images is systematically influenced by sentence syntax and the language model used, we fitted a mixed-effects logistic regression model. The binary outcome variable was agent position (0 = left, 1 = right). Incorrect drawings or drawings with other agent positions were excluded. The fixed effects were syntax (active vs. passive), model (FLUX vs. DALL⋅E 3), and their interaction. Sentence was modeled as a random intercept to account for item-level variability. The model was fitted using the glmer() function from the lme4 package (version 1.1-37; R version 4.5.1, [38]), with a binomial link function.

Model fit statistics are reported in Table 3, indicating an AIC of 4695.8 and a log-likelihood of -2342.9. Scaled residuals ranged between -1.89 and 2.04, suggesting an acceptable model fit.

**Table 3:** Model fit statistics and scaled residuals for the agent position model.

Model fit	
AIC	4695.8
BIC	4727.0
Log likelihood	–2342.9
Deviance	4685.8
Residual DF	3787
**Scaled residuals**	
Min	–1.8943
1st quartile	–0.6919
Median	–0.5526
3rd quartile	0.8585
Max	2.0378

Table 4 summarizes the random effects. Including a random intercept for sentence (variance = 0.079) accounting for item-level variability. The intraclass correlation coefficient (ICC) was 0.02, indicating that only 2% of the variance in agent placement was attributable to sentence-level differences.

**Table 4:** Random effects of agent position model.

Groups	Name	Variance/Std. Dev.
sentence	(Intercept)	0.07901/0.2811
Number of observations	3792	
Number of sentence groups	10	

Table 5 displays the fixed-effect estimates. There was a significant main effect of the variable syntax: passive sentences were more likely to place the agent on the right compared to active sentences (OR = 2.83, 95% CI = [2.34, 3.43], *p* < .001). This suggests that passive constructions strongly influence spatial representation. The main effect of the variable model was not significant (OR = 0.95, 95% CI = [0.78, 1.17], *p* = 0.632), indicating no overall difference in agent placement between FLUX and DALL⋅E 3.

**Table 5:** Fixed effects estimates for agent position model.

	Estimate	Std. Error	z value	Pr(>|z|)
(Intercept)	–0.90506	0.11465	–7.894	$2.93\times 10^{-15}$ ***
Model	–0.04951	0.10329	–0.479	0.632
Syntax	1.04171	0.09734	10.702	$<2\times 10^{-16}$ ***
Model:syntax	0.81295	0.14166	5.739	$9.54\times 10^{-9}$ ***
**Correlation of fixed effects**				
	**(Intercept)**	**Model**	**Syntax**	
Model	–0.441			
Syntax	–0.470	0.520		
Model:syntax	0.321	–0.729	–0.685	

*Note* In this table and elsewhere, *** indicates *p* < 0.001, denoting a highly significant result.

A significant interaction between the variables model and syntax (OR = 2.25, 95% CI = [1.71, 2.97], *p* < .001) showed that the impact of syntax on agent positioning differed across models, with FLUX exhibiting a stronger leftward bias in active constructions than DALL⋅E 3, whereas in passive constructions both models showed a similarly strong rightward bias.

The marginal *R*^2^ was 0.150, indicating that the fixed effects explained 15% of the variance in agent placement. The conditional *R*^2^ was 0.170, suggesting that the full model (including random effects) explained 17% of the variance. These values were computed using the r.squaredGLMM() function from the MuMIn package.

These findings indicate that sentence syntax has a robust influence on spatial representation in AI-generated images, with passive constructions leading to more frequent rightward agent placement. Although both AI models responded to syntactic cues, DALL⋅E 3 exhibited a stronger syntactic bias than FLUX. Random intercepts for sentence accounted for minimal additional variance, suggesting that semantic effects were observable but small, and that the observed effects were primarily driven by syntax and model differences.

## Multinomial model

While the initial analysis treated “agent position” as a binary variable (left vs. right), a more nuanced classification of AI-generated outputs is possible. In 25 cases (0.63% of the total), the agent was positioned in a different spatial relation (e.g., above, below, behind, or in front) rather than to the left or right of the other figure. In another 183 cases (4.6%), the images generated did not reflect the semantics of the prompt correctly. To account for these additional outcomes, we extended the analysis using a multinomial model, which treats agent position as a categorical dependent variable with four possible outcomes: left placement (0), right placement (1), other placement (2), and semantically incorrect content (3).

The multinomial model employed a simpler structure than the initial binary logistic regression, as it did not include random effects. This choice was motivated by the limited number of cases in the ‘other’ category (n = 25), which led to convergence issues and rendered the estimation of random effects unreliable. A key advantage of the multinomial model, however, is its ability to accommodate four unordered outcome categories, while remaining relatively easy to interpret. The model compares each of the three less frequent agent positions to a reference category (left placement) using separate logistic regressions, estimating the log-odds of each alternative relative to the reference. The model was fitted using the nnet::multinom() function, which provides model-based (naive) standard errors under the assumption of independent observations. While our data include within-prompt dependencies, the assumption of independent observations is unlikely to be fully satisfied. However, the repeated image generations by the AI models can be considered more independent from one another than would be the case with human participants, where earlier drawings might influence subsequent ones. As robust or cluster-adjusted standard errors are not supported by this implementation, the reported *p*-values should be interpreted with caution.

By incorporating the two additional categories for agent position (other and incorrect content), this second analysis provides a richer understanding of AI-generated spatial positioning, revealing not only how agent placement shifts in response to syntax and model differences but also whether these factors influence incorrect or unconventional outputs. This approach ensures that drawings with unconventional perspectives or incorrect content are not treated as missing data but rather as meaningful outcomes that can inform the interpretation of AI behavior. The multinomial logistic regression was specified as (agent_position~model+syntax), where agent_position was a nominal outcome variable with four unordered categories (left, right, other, incorrect), and model and syntax were treated as categorical predictors with two levels each. The model was fitted using the multinom() function from the nnet package (version 7.3-20; R version 4.5.1, R Core Team, 2025). The model used a generalized logit link function appropriate for nominal outcome variables to assess the influence of model and syntax on the agent position. The dependent variable had four unordered categories (left, right, other and incorrect), and the reference category was set to “left”. The model estimated a total of 16 parameters, including intercepts and effects of two categorical predictors (model and syntax), each with two levels. The model converged after 30 iterations with a final log-likelihood of 3241.10 (Residual Deviance = 6482.20; AIC = 6500.20).

The model showed significant effects for both predictors in several contrasts. Passive syntax significantly increased the likelihood of agents being placed on the right (*β* = 1.42, SE = 0.07, z = 20.28, p < .001) and in the “other” position (*β* = 1.14, SE = 0.42, z = 2.71, p = .007), compared to the left. The model also significantly differed across AI models in multiple contrasts, see Table 6 for full results. Since they are only 25 cases of other positioning of the agent in the database, the standard errors for the “other” category are clearly larger. While the model estimates for the ‘other’ category are interpretable, the small number of observations (n = 25) leads to wider confidence intervals and reduced statistical power. Therefore, conclusions regarding this category should be drawn with caution.

**Table 6:** Multinomial logistic regression predicting agent position (Reference Category: Left) Odds Ratios (ORs) are reported with 95% confidence intervals. Significance: *p < .05; **p < .01; ***p < .001.

Outcome	Predictor	β	SE	z	*p*	OR [95% CI]	Sig.
Right (1)	Intercept	–1.10	0.06	–17.45	< .001	0.33 [0.29, 0.37]	***
	Model	0.37	0.07	5.30	< .001	1.44 [1.25, 1.66]	***
	Syntax	1.42	0.07	20.28	< .001	4.12 [3.58, 4.74]	***
Other (2)	Intercept	–4.41	0.34	–13.06	< .001	0.012 [0.006, 0.024]	***
	Model	–2.98	1.02	–2.92	.0035	0.051 [0.007, 0.38]	**
	Syntax	1.14	0.42	2.71	.0067	3.13 [1.37, 7.15]	**
Incorrect (3)	Intercept	–2.81	0.14	–20.15	< .001	0.060 [0.046, 0.078]	***
	Model	0.76	0.16	4.75	< .001	2.14 [1.58, 2.89]	***
	Syntax	–0.10	0.16	–0.59	.5534	0.91 [0.67, 1.23]	

In the multinomial regression model predicting agent position, several meaningful patterns emerged. Compared to the reference category (left placement of the agent), the odds of the agent being placed on the right were significantly increased by passive syntax (OR = 4.12), suggesting that passive constructions strongly promote a rightward shift in agent placement. Outputs from FLUX also modestly increased the odds of right positioning of the agent relative to DALL⋅E 3 (OR = 1.44). In contrast, “other” positioning of the agent (above or in front) was markedly less likely overall (OR = 0.012 for the intercept), and was nearly eliminated by FLUX (OR = 0.051), despite being more likely in passive constructions (OR = 3.13). The “incorrect” images category was also relatively rare (OR = 0.060 for the intercept), but its likelihood was significantly increased by FLUX (OR = 2.15), with no significant influence of syntax (OR = 0.91). In summary, passive syntax is the strongest predictor of rightward agent positioning, whereas model architecture primarily affects the likelihood of producing rare or unconventional agent placements, including semantically incorrect depictions.

In summary, passive syntax shifts agent positioning to the right, with this effect amplified by the AI model. The odds ratios highlight the robust role of syntax in shaping spatial positioning, while the model’s influence is particularly evident in the “other” category. One limitation of this second analysis is that the multinomial model did not account for potential clustering or repeated structures (e.g., by sentence), due to the limitations of the nnet::multinom() implementation. As a result, standard errors may be underestimated, and *p*-values could be overly optimistic.

### Complementary insights of the two models

To provide an initial overview of the data patterns, we first summarize the descriptive trends in agent positioning across models and syntactic conditions prior to statistical analysis. For DALL⋅E 3, agent placement shows a strong rightward asymetry in passive sentences, whereas in active sentences the placement of the agent is comparatively balanced, with no clear right or left preference. FLUX, in comparison, shows pronounced asymmetries in both conditions: agents are disproportionally positioned on the right in passive sentences and on the left in active sentences. This pattern suggests that DALL⋅E 3 displays only weak sensitivity to syntactic voice in spatial agent positioning, whereas FLUX systematically adjusts placement according to sentence structure. While both AI-models show some left–right reversal between active and passive constructions, the effect substantially stronger for FLUX, indicating a higher sensitivity for syntactic structure. DALL⋅E 3 exhibits a mild shift rather than a categorical change, implying limited syntactic sensitivity in how it maps agent roles onto spatial layouts.

The binary mixed-effects logistic regression and the multinomial regression model offer complementary insights. The mixed-effects model focused on a reduced dataset that excluded ambiguous or incorrect outputs, yielding a cleaner estimate of how syntax and model interact to affect left–right positioning. By including a random intercept for sentence, it accounted for item-level variability and provided robust inference within the binary coding scheme. The multinomial model, in contrast, incorporated all four observed outcomes (left, right, other, incorrect), allowing us to examine not only canonical positioning but also atypical or error-prone outputs. Because this model lacked random effects and assumed independence, its *p*-values should be interpreted with caution, particularly for the underrepresented “other” category.

The binary model confirmed a strong syntactic effect, showing that passive constructions increased the likelihood of rightward placement. The multinomial model reaffirmed this finding and additionally highlighted model-specific differences in the likelihood of generating “other” or incorrect outputs. Together, the models show that syntax robustly shifts agent placement to the right, while model architecture modulates the frequency of unconventional outcomes.

We report AIC values for transparency, but stress that they are not directly comparable: the two models are fitted to different outcome structures (binary vs. multinomial) and rely on distinct likelihood functions. The more meaningful contrast lies in their research focus: the binary model provides controlled inference on canonical left–right placement, whereas the multinomial model offers a descriptive account of the full distribution of outcomes. Although the multinomial model explained only a modest proportion of variance, such values are common in categorical behavioral data, and the observed syntactic effects remain theoretically meaningful.

In summary, the binary model isolates predictors of canonical left–right positioning with strong statistical control, while the multinomial model captures the full distribution of outcomes, including errors and atypical placements. Despite their methodological differences, both approaches converge on the same key finding: syntactic structure strongly influences agent positioning, with passive constructions driving rightward placement, and the two AI systems differing in the strength and consistency of this effect.

## Discussion

The present study examined how two current text-to-image AI models, DALL⋅E 3 and FLUX, handle spatial agency when interpreting simple sentences with two animate entities in active and passive voice. Our primary aim was to investigate whether these models exhibit human-like spatial biases in visual representation, particularly the left-to-right agent placement bias that has been robustly observed in Western literate populations.

To investigate whether syntactic structure and semantic content influence the spatial placement of agents, we created a database of images generated by the FLUX and DALL⋅E 3 AI models. Our hypothesis was that active constructions, which in human-produced images tend to favor leftward positioning in left-to-right reading languages, would yield similarly left-placed agents in AI-generated outputs. By comparing outputs across different AI models, we identified systematic differences in how they encode spatial relations.

To balance statistical rigor with interpretability, we limited our analysis to two models: a binary logistic regression and a multinomial logistic regression. The binary model was selected for its ability to test a theoretically central contrast: whether agent positioning shifts systematically to the right in response to passive syntax, while accounting for sentence-level variability via random intercepts. This model allowed us to isolate the effect of syntactic structure and AI model type on a clear spatial binary (left vs. right agent positioning), which directly maps onto established psycholinguistic theories of thematic role assignment and canonical agent-patient ordering in the spatial layout. However, since a subset of the generated images showed agent placements that deviated from the left–right binary or failed to depict the prompt content accurately, a second model was necessary to capture the full outcome structure.

The multinomial model allowed us to include all four observed categories of agent positioning (including other unconventional placements and drawings with incorrect semantic content), thus avoiding data loss or overly simplistic dichotomization. Although this model does not include random effects and assumes independence of observations, it provides a more ecologically valid and comprehensive representation of the variability in AI-generated outputs. Taken together, these two modeling approaches offer complementary perspectives: the binary model provides controlled inferential testing of a theoretically motivated contrast, while the multinomial model descriptively captures the broader distributional landscape of spatial behavior across models and syntactic frames.

The main finding from our analysis is that syntactic structure plays a substantially larger role than semantic content in determining agent placement in the generated images. This pattern mirrors prior findings from human behavioral studies, including our previous cross-linguistic study with Spanish, German, and Czech participants, where we found similar patterns regarding the spatial agency bias among speakers of these three left-to-right languages [33]. The present results extend those findings to AI models: even when sentence content is held constant, syntactic voice (active vs. passive) systematically influenced spatial layout, though this effect was not identical across models: The interaction between the variables model and syntax was significant, meaning that the two AI systems exhibit distinct patterns of spatial bias. FLUX consistently placed agents on the left in active sentences and on the right in passive ones, indicating strong syntactic asymmetries in both directions. This behavior partially mirrors human spatial agency preferences in active constructions but departs sharply in passive constructions, where humans tend to show little to no bias. DALL⋅E 3, in comparison, showed only a weak asymmetry in active sentences (barely over 50%) and instead showed a stronger, rightward asymmetry in passive sentences. Overall, DALL⋅E 3 placed agents on the right more frequently than either human participants or FLUX.

These differences suggest that while both models exhibit systematic spatial behavior, their internal mappings between linguistic structure and spatial composition diverge from each other but also from human cognition. From the two investigated models, FLUX seems to more closely approximate the output of a human Westerner, particularly in active constructions. From a psycholinguistic point of view, the AI models seem to be responding more to the Advantage of the First Mention than to the Spatial Agency Bias (since in the passive sentences both models show a clear tendency to draw the agent, mentioned second, to the right).

Lastly, our study aligns with previous research on other biases in AI-generated art, such as gender and racial biases (e.g., [2,3]). Specifically, we also found that these models produce images that systematically differ from those created by humans. Importantly, such biases become detectable only when a sufficiently large and structured dataset is available, allowing for measurable and systematic analysis.

### Limitations

Despite the significant effects observed for syntax and AI model in both analyses, the explained variance was relatively low, as reflected by the marginal *R*^2^ of 0.15 and pseudo-*R*^2^ of 0.17 in the mixed-effects model. These values indicate that only a small portion of the variability in agent positioning can be attributed to the fixed and random effects included in the model. Similarly, the multinomial model, while descriptive of outcome patterns, did not report variance explained, and likely shares this limitation due to its simplified structure and lack of control for clustering. Low *R*^2^ values are not uncommon in behavioral data, especially when modeling outputs from large AI systems that integrate complex and opaque generative processes. Nonetheless, the limited explanatory power of our models suggests that additional factors (such as sentence semantics, latent model biases, or training data artifacts) likely influence agent positioning but were not captured in the present analysis. Furthermore, the observed effects, though statistically significant, may only account for broad tendencies rather than strong, deterministic shifts. Therefore, the findings should be interpreted as indicative of probabilistic patterns rather than precise predictive mechanisms. Future work might improve model fit by incorporating richer semantic predictors, image-based features, or more fine-grained representations of AI architecture and training parameters. Additionally, hierarchical modeling frameworks that allow for more flexible random structures could offer better insight into sentence-level variability and dependencies across stimuli.

Another indicator of model limitations is the relatively high frequency of semantically wrong responses (cases in which the model drew something other than the prompt), coded in this database as “incorrect”. In both models, these rates exceeded those found in human data, highlighting that even cutting-edge AI still struggles to interpret and visualize prompts reliably. Since these two models are not behaving the same, we have to be careful to not overgeneralize our results to other AI models not yet studied.

Despite these limitations, the statistical models presented here offer meaningful and theoretically grounded insights into how linguistic structure and AI architecture jointly shape spatial representation in image generation. The observed effects of syntax were consistent across modeling approaches and robust in direction and magnitude, aligning with known cognitive and linguistic patterns in human spatial encoding. The inclusion of both binary and multinomial analyses provided complementary perspectives: one offering statistical rigor and controlled inference, and the other capturing a broader range of outcomes, including deviations and errors, which are essential for understanding the full behavioral spectrum of generative AI systems. Thus, even in the presence of unexplained variance, the current findings contribute valuable evidence for systematic, linguistically driven behavior in AI-generated visual outputs and lay the groundwork for more fine-grained, multimodal modeling in future work.

### Implications

Our findings point to broader implications regarding cultural and cognitive biases in AI-generated visual content. While the models were not explicitly trained to produce spatial asymmetries linked to reading direction, their output shows traces of such biases. This suggest that the training data, overwhelmingly drawn from Western sources, implicitly shaped these behaviors. The piloting before the main data collection for this study showed that when prompted in non-English languages, the models either defaulted to English (DALL⋅E 3) or failed to interpret the prompt correctly (FLUX), indicating a strong anglocentric orientation in their current capabilities.

This raises a critical concern: as AI-generated imagery becomes increasingly ubiquitous online, it may amplify and normalize Western-specific visual conventions, subtly diminishing culturally diverse patterns of representation. As previous research has shown, spatial biases differ across languages and cultures [1,12,13]. The homogenizing influence of AI could thus erode forms of cognitive diversity that are currently underrepresented but no less important.

There is a growing awareness of visible biases in AI art, such as the under-representation of non-normative body types or the stereotyping of ethnic identities. Our study shows that more subtle cognitive and/or linguistic features such as the Spatial Agency Bias can also shift under AI influence. Because these features operate below conscious awareness, we may not even notice what is being lost. Our study highlights that if one of the expectations of AI-generated art is to mimic human art, it does not (yet) do so accurately. More precisely, in its current form, AI-generated images align more closely with the biases found in Western artistic traditions, rather than reflecting the diversity and variability that characterize human creative expression as a whole.

## Conclusion and outlook

To our knowledge, this is the first study to investigate the influence of reading-writing direction and syntactic structure on spatial composition in AI-generated images. By systematically testing two high-performing models with controlled linguistic prompts, we showed that these AI models sometimes show spatial biases which reflect human cognition, while other times deviate from the expected output for humans quite considerably.

We provide all data and analysis scripts in an open repository to facilitate replication and longitudinal comparisons as new models emerge. As text-to-image generation becomes increasingly integrated into digital communication and artistic production, it will become relevant to be able to track how these spatial biases evolved over time.

Future research should replicate this research in languages with different script directions (e.g., Hebrew, Arabic) once models demonstrate sufficient comprehension of these less widespread languages. The method presented in our article is fully replicable and has proven sufficient to uncover both the spatial agency bias and the advantage of first mentioned. Working with APIs enables precise control over prompting, which will be crucial when conducting studies in languages other than English. Such cross-linguistic studies will be vital for understanding how and whether AI models can represent the full diversity of human spatial cognition in art.
